# Geographical Environment Factors and Risk Assessment of Tick-Borne Encephalitis in Hulunbuir, Northeastern China

**DOI:** 10.3390/ijerph14060569

**Published:** 2017-05-26

**Authors:** Yifan Li, Juanle Wang, Mengxu Gao, Liqun Fang, Changhua Liu, Xin Lyu, Yongqing Bai, Qiang Zhao, Hairong Li, Hongjie Yu, Wuchun Cao, Liqiang Feng, Yanjun Wang, Bin Zhang

**Affiliations:** 1 State Key Laboratory of Resources and Environmental Information System, Institute of Geographic Sciences and Natural Resources Research, CAS, Beijing 100101, China; lyf@qdio.ac.cn (Y.L.); gaomx@igsnrr.ac.cn (M.G.); lx@lreis.ac.cn (X.L.); baiyq@lreis.ac.cn (Y.B.); zhaoq@lreis.ac.cn (Q.Z.); lihr@igsnrr.ac.cn (H.L.); 2 Institute of Oceanology, Chinese Academy of Sciences, Qingdao 266071, China; lch@qdio.ac.cn (C.L.); fenglq@qdio.ac.cn (L.F.); yjwang@qdio.ac.cn (Y.W.); zhangbin@qdio.ac.cn (B.Z.); 3 Jiangsu Center for Collaborative Innovation in Geographical Information Resource Development and Application, Nanjing 210023, China; 4 National Science and Technology Infrastructure Center, Beijing 100862, China; 5 State Key Laboratory of Pathogen and Biosecurity, Beijing Institute of Microbiology and Epidemiology, Beijing 100071, China; fang_lq@163.com (L.F.); caowc@bmi.ac.cn (W.C.); 6 University of Chinese Academy of Sciences, Beijing 100049, China; 7 School of Public Health, Fudan University, Key Laboratory of Public Health Safety, Ministry of Education, Shanghai 200032, China; cfetpyhj@vip.sina.com

**Keywords:** tick-borne encephalitis, geographic and environmental factors, spatial autocorrelation, geographic weighted regression

## Abstract

Tick-borne encephalitis (TBE) is one of natural foci diseases transmitted by ticks. Its distribution and transmission are closely related to geographic and environmental factors. Identification of environmental determinates of TBE is of great importance to understanding the general distribution of existing and potential TBE natural foci. Hulunbuir, one of the most severe endemic areas of the disease, is selected as the study area. Statistical analysis, global and local spatial autocorrelation analysis, and regression methods were applied to detect the spatiotemporal characteristics, compare the impact degree of associated factors, and model the risk distribution using the heterogeneity. The statistical analysis of gridded geographic and environmental factors and TBE incidence show that the TBE patients mainly occurred during spring and summer and that there is a significant positive spatial autocorrelation between the distribution of TBE cases and environmental characteristics. The impact degree of these factors on TBE risks has the following descending order: temperature, relative humidity, vegetation coverage, precipitation and topography. A high-risk area with a triangle shape was determined in the central part of Hulunbuir; the low-risk area is located in the two belts next to the outside edge of the central triangle. The TBE risk distribution revealed that the impact of the geographic factors changed depending on the heterogeneity.

## 1. Introduction

Tick-borne encephalitis (TBE) is a human viral infectious disease involving the central nervous system. Over the past decades, TBE has become a growing public health concern in Western Europe, Siberia, and northeastern China [1]. Ticks, the main vector of the TBE virus, are considered to be one of the most important vectors of natural focus diseases affecting livestock, humans, and companion animals. Approximately 10% of the currently known 867 tick species act as vectors of a broad range of pathogens [1]. The distribution of TBE is closely related to geographic and environmental factors [2]. Therefore, it is necessary to know the existing and potential natural foci of TBE and determine the relative environmental factors of high TBE risks.

**Figure 1:**
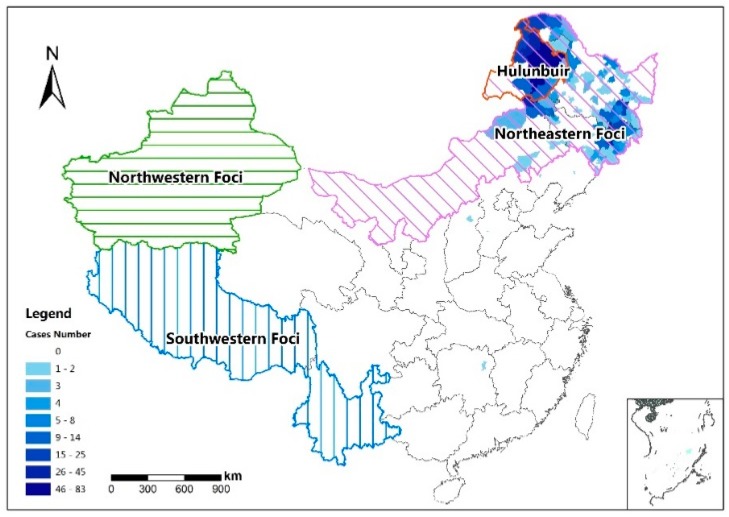
Major tick-borne encephalitis (TBE) foci in China. The background is the reported TBE case number on a county level in 2011.

The most important vector of TBE is *Ixodes persulcatus* in China, while *Ixodes ricinus* is more common in Europe [3]. The human TBE was first reported in the forested areas of northeastern China in 1943 [4,5]. After the foundation of the P. R. China, deforestation operations were extended from winter to the whole year. The numbers of TBE cases in forest districts sharply increased from 1951 to 1953. Subsequently, the numbers decreased notably due to the planned control and intervention such as vaccination and health education by local governments [4]. As shown in Figure 1, the distribution of TBE natural foci in China can be divided into three parts: northeastern, northwestern and southwestern foci [6]. The northeastern area provides suitable habitats for the main vectors and hosts of the TBE virus, making it the main focus of TBE. It can be further divided into the Changbai Mountains, Greater Khingan Range, and Lesser Khingan Range infectious foci. The northwestern foci area, which is located in the Xinjiang Province, has fewer cases reported than the northeastern natural foci. It comprises the Tian Shan and Altai Mountains infectious foci. The southwestern part of China has also been considered as suspicious infectious focus because the TBE virus has been detected here [7]. The epidemiological study of TBE showed that the TBE risk still existed in the originally defined natural foci between 1992 and 1994 [8]. From 1991 to 2002, the number of TBE cases in this area increased continuously and the risk area expanded gradually. People attributed the high TBE incidences to the lack of immunity and the insufficient vaccination coverage [9]. Various infectious diseases have been reported since 2006 through the China Centers for Disease Control’s (CDC) direct network report system. The largest number of TBE cases between 2006 and 2013 was in 2011. In this year, 576 cases were reported from Northeast China, accounting for 99.14% of the total number. Among them, 214 TBE cases were reported in Hulunbuir, making it one of the most severe endemic areas of TBE in mainland China. 

Based on previous studies of the types of tick-borne diseases, such as TBE, Lyme disease, tick-borne relapsing fever, and Ehrlichiosis, the related geographic and environmental factors mainly include terrain and topography, climate, and soil and vegetation [10,11,12]:

(1) The topographic factors include elevation, slope, and aspect. These factors play important roles in the distribution of ticks and their hosts by affecting the reallocation of the hydrothermal combination. Merler used classification tree method to analyze the distribution of *Ixodes ricinus* in Trentino, Italian Alps, and concluded that the most important factors determining the distribution of the ticks are the altitude and geological environment [13]. Toomer used the digital elevation model (DEM) and other factors to simulate the general distribution of Pan-African ticks [14]. Randolph et al. used the DEM and Land Surface Temperature (LST) as predictive variables and reported that the distribution of five tick aggregation places in central Europe and around the Baltic Ocean is closely related to the DEM [15]. Materna et al. mentioned in their study that the density of ticks in small-scale research is highly impacted by the aspect [16].
(2) Climate factors, such as temperature, light duration, and rainfall, determine the living range of hosts and vectors to a certain extent, which affects the distribution of natural foci. Lindgren suggested that more ticks might survive in a mild winter in host and reservoir animals [17]. Due to an early arrival of the spring and/or late arrival of the next winter, ticks will be active for an extended period. Eisen reported that the tick density is closely related to the daily maximum temperatures [18]. Süss et al. proposed that an increase in the temperature up to a certain level causes the acceleration and extension of the developmental cycle of the ticks, increase in the egg production and population density, and shift of the risk areas [19]. Kahl et al. concluded that the relative humidity (RH) affects the life circle of ticks due to the transformation and absorption of water vapor in half-saturated air [20].
(3) The vegetation can provide a suitable living environment for ticks and their vectors. The density of ticks correlates to the type and structure of the forest; it is the highest in mixed and deciduous forests [15]. Jackson reported that the Lyme disease incidences in 12 Maryland counties were the highest when the edge-contrast index of the forest–herbaceous edge reached 53% [21].

Although the relationships between these geographic and environmental factors have been studied in some papers [4,8,9,22], the factors and risk distribution of TBE in China is unclear. Most domestic studies focused on the last century and were based on data with coarse spatial resolution and incomplete statistic records. In this paper, we will assess the distribution of TBE risks and relatively geographical environment factors in Hulunbuir, China. The specific goals of the research are to: (1) characterize the spatiotemporal distribution of TBE epidemics; (2) compare the relative importance of different geographic and environmental factors for the TBE risks; and (3) identify the TBE risk distribution and spatial variability of the regression model factors.

## 2. Material and Methods

### 2.1. Study Area

**Figure 2:**
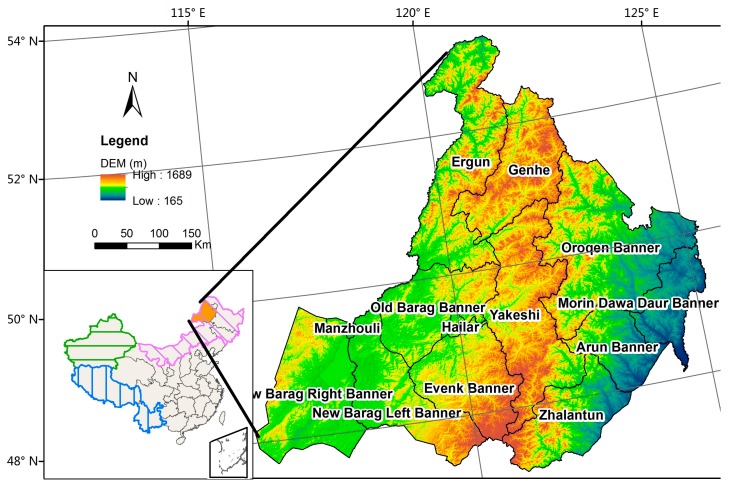
Map of the study area with digital elevation models in the background.

Hulunbuir is a prefecture-level city in Inner Mongolia, northeastern China (115°31′–125°04′ E, 47°05′–53°20′ N). The area of Hulunbuir comprises ~264,000 km^2^. It contains 13 different county-level jurisdictions, one district, five county-level cities, four banners, and three autonomous banners (Figure 2), and includes 159 towns and sumus (sumu is a unique type of town in Inner Mongolia). The Greater Khingan Range, extending roughly from north to south, divides Hulunbuir into three parts. The eastern part comprises a broad area of low hills and valley plains with an average altitude of 400 m and includes Zhalantun, Arun Banner, and Morin Dawa Daur Autonomous Banner. This part consists of large areas of farmland with high outputs of soybean, corn, and rice. The central part mostly contains boundless forests of the Greater Khingan Range at an average altitude of ~850 m, including Yakeshi, Genhe, and Oroqen Autonomous Banner. The main forest tree species include Larix larch, birch, and *Pinus sylvestris*. The western part of Hulunbuir is mainly covered with grassland and pastures at an average altitude of ~560 m, including the New Barag Right Banner, New Barag Left Banner, and Evenk Autonomous Banner. 

### 2.2. Data and Preprocessing

#### 2.2.1. Disease Data

All TBE cases reported from January 2006 to December 2013 in Hulunbuir were obtained from the Beijing Institute of Microbiology and Epidemiology. The recorded identity information was thoroughly checked. In total, 680 TBE cases were registered in Hulunbuir from 2006 to 2013. Almost all the TBE disease cases were georeferenced by the places of patients’ residence. We excluded five cases which were non-native in our study.

**Figure 3:**
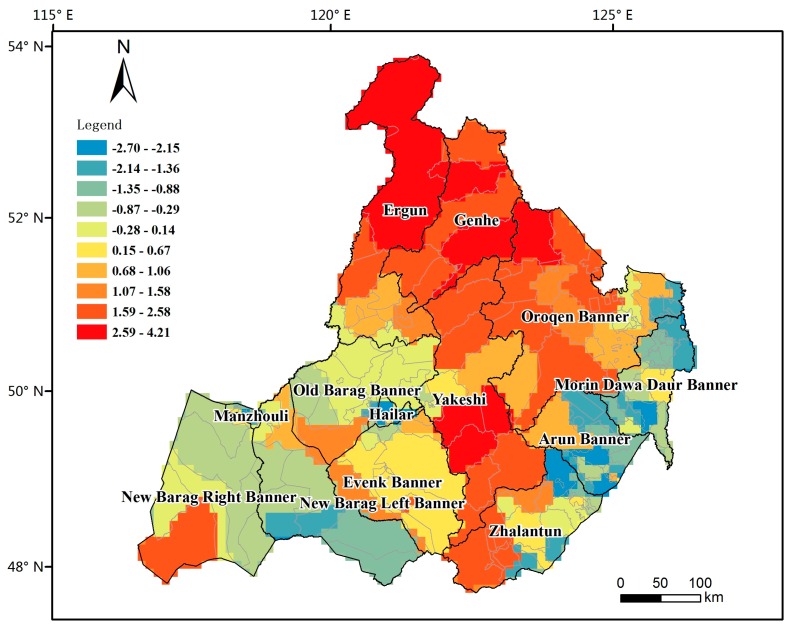
The logarithmic smoothed tick-borne encephalitis (TBE) incidences. The background is characterized by the logarithmic smoothed TBE incidence values, which were used as dependent variable in the regression models.

The TBE incidences were derived from the TBE cases and population. The population statistics are based on the Population Census of the People’s Republic of China in 2010 [23]. Because the annual TBE incidence number would also be subjected to the high variance of short-term observations [24]. In this paper, we used the average yearly incidences from 2006 to 2013 to reduce the variance of the rates. The differences in the population size led to variance instability and spurious outliers when the raw rates were used to estimate the underlying risk. The Empirical Bayes Smoothing (EBS) method in GeoDa (https://geodacenter.asu.edu/) was utilized in this study to smooth the TBE raw incidences. Because the distribution curve of the smoothed rates is still seriously right-skewed. The logarithmic value of the EBS-smoothed TBE incidence was used as the dependent variable in the following regression models to compare the results of different transforming methods. The logarithmic smoothed TBE incidence is shown in Figure 3.

#### 2.2.2. Geographic and Environmental Data

The Shuttle Radar Topography Mission (SRTM) DEM with a spatial resolution of 90 m was downloaded from the National Aeronautics and Space Administration (NASA). The slope and aspect data of the DEM were transformed using ArcGIS tools (www.arcgis.com). 

The Normalized Difference Vegetation Index (NDVI) and Enhanced Vegetation Index (EVI) were selected from MOD13A3. Global MOD13A3 data were provided monthly at 1-km spatial resolution as a gridded level-3 product in the sinusoidal projection, which was downloaded from NASA’s Earth Observing System Data and Information System (EOSDIS). The MODIS Projection Tool (MRT) was used to project and mosaic the MOD13A3 images during the image processing. Because MOD13A3 images are monthly data, we used several batch programs in Python to calculate the annual average value. 

The climate data were downloaded from the China Meteorological Data Sharing Service System (http://cdc.nmic.cn/). To obtain better interpolation results for the whole study area, 26 climate sites were selected in a 160-km range around the boundary of Hulunbuir. Five climate indices have been included in the study such as the average annual temperature, average annual precipitation and rainfall depth, precipitation frequency and number of days with at least 0.1 mm of daily precipitation, average relatively humidity, and daily average sun hours.

Grid sets were used to link the TBE incidence data with geographic factors. Grid discretization was used to refine the resolution of the raw features, detect and trace the relationship between spatial attributes on a more elaborate scale, and reduce the constraints between administrative boundaries. Referring to the incremental spatial autocorrelation results mentioned in Section 2.3.1, with the distance of 106,818.21 m, the average number of TBE cases were most likely to be spatially aggregated. The regression experiments in this paper were performed based on 10 km × 10 km grids; the whole grid set comprises 2340 grids. The grid units were assigned based on the mean values of covered features. In a similar research of Merler, doing samples in 99 sites over an area of approximately 2700 km^2^, was evidenced to get effective results in identifying the mesoscale areas at greater potential risk with a relatively low sampling effort [13]. The chosen spatial resolution of 10 km × 10 km was appropriate in this paper.

### 2.3. Methods

Statistical methods were used to detect the seasonal and population characteristics of TBE infection. Global and local autocorrelation analyses were used to detect the spatial and yearly temporal distribution characteristics of TBE in the study area. Global regression analysis was used to rank the impact of all involved environmental variables. Local analysis was carried out using local regression to explore the spatial variability of the parameters of the regression models and model the distribution of the TBE risks depending on the heterogeneity.

#### 2.3.1. Spatial Autocorrelation

(1) Global Autocorrelation

The global Moran’s *I* was used to obtain the spatial distribution of the TBE rates throughout the study area by measuring the approximation of the observations at nearby locations. The Moran’s *I* is calculated as follows:

(1) $$I=\frac{\sum_{i=1}^{n}\sum_{j=1}^{m}w_{ij}(x_{i}-\bar{x})(x_{j}-\bar{x})}{s^{2}\sum_{i=1}^{n}\sum_{j=1}^{m}w_{ij}}$$

where *x_i_* and *x_j_* represent the TBE case numbers of town *i* and town *j*, respectively; $\bar{x}$ is the mean case number of all towns; ***S***^2^ is the variance of the number of TBE cases, *n* is the number of towns in Hulunbuir; *m* is the number of towns, which are within a certain distance of town *i*; *w_ij_* is the weight ratio of town *j* to town *i*, when town *i* is contiguous to town *j* and the weight *w_ij_* is 1; otherwise, the weight *w_ij_* is 0. 

In this paper, we used incremental spatial autocorrelation method to get *Z*-scores of 8 years’ average TBE case numbers with a series of distance. The result showed that *Z*-score reached the peak value with the distance of 106,818.21 m, which indicated that, with this distance, the average number of TBE cases was most likely to be spatially aggregated. Therefore, the certain distance mentioned above was set to 106,818.21 m.

The autocorrelation feature is determined by the expectation value, *E(I)*, in the random mode. If the spatial autocorrelation is positive, the Moran’s *I* is greater than *E(I)*. If the spatial autocorrelation is negative, the Moran’s *I* is smaller than *E(I)*. In addition, the *Z*-score represents the possibility of random spatial distribution [25].

(2) Local Autocorrelation

Local autocorrelation indices can further reveal the similarities and correlations of adjacent units and can be used to detect “hotspots” as well as the heterogeneity of the data. To indicate, whether there is a cluster of high or low TBE rates, this paper chose Getis-Ord Gi. The equation is as follows [26]:

(2) $$G_{i}^{*}=\frac{\sum_{j=1}^{n}\omega_{i,j}x_{j}-\bar{X}\sum_{j=1}^{n}\omega_{i,j}}{S\sqrt{\frac{\left[n\sum_{j=1}^{n}\omega_{i,j}^{2}-{\left(\sum_{j=1}^{n}\omega_{i,j}\right)}^{2}\right]}{n-1}}}$$

where *x_j_* is the TBE case number of feature *j*; *ω_ij_* is the spatial weight between feature *i* and *j*, *ω_ij_* = 1 if the regions i and j are within a distance of 106,818.21 m, set in global autocorrelation, and *ω_ij_* = 0 otherwise; *n* is the total number of towns in Hulunbuir; and $\bar{X}$ and *S* are the mean value and variance of the number of TBE cases, respectively. 

The G_i_* statistic provides a *Z*-score for each town in Hulunbuir. With respect to statistically significant *Z*-scores, the larger the *Z*-score is, the more intense is the clustering of high values (“hot spot”). With respect to significant negative *Z*-scores, the smaller the *Z*-score is, the stronger is the clustering of low values (“cold spot”).

#### 2.3.2. Spatial Regression

(1) Global Multiple Linear Regression

Multiple linear regression (MLR) was used as global regression model to rank the correlations between the environmental independent elements and TBE risks. The general MLR is as follows:

(3) $$y_{i}=\beta_{0}+\sum_{i=1}^{n}\left(\beta_{i}x_{i}+\varepsilon_{i}\right)\;\left(i=1,\ldots ,n,\right)$$

where *x_i_*, *y*, *β*_0_, and *β_i_* are the environmental factors, logarithmic smoothed TBE rates, intercept, and partial regression coefficient, respectively, and ε is the random error assuming that $\varepsilon_{i}\sim N(0,\sigma^{2})$. Ordinary least squares (OLS) are used to estimate the regression parameters. 

Stepwise regression was used in the modeling process to obtain the most suitable model. The R^2^ value was computed for each model to assess the goodness-of-fit. 

(2) Local Geographic Weighted Regression

With respect to the TBE risks with spatial heterogeneity, Geographic Weighted Regression (GWR) was used to model the TBE risks based on the global statistics analysis results. The factors selected from the MLR were tested before the modeling using statistical correlation analysis.

The weights in the GWR method are the functioned distances between the estimated points and other observation points. The parameters depending on the different spatial location can be used to determine the geographic variation of the different factors. The general form of the GWR is as follows [27,28]:

(4) $$y_{i}=a_{0}(u_{i},v_{i})+\sum_{k}a_{k}(u_{i},v_{i})x_{ik}+\varepsilon_{i}$$

where *y_i_* is the dependent variable at the point *i*; *x_ik_* is the value of the *k*-th independent variable at the point *i*, where *i* is the number of sample points and *k* is the number of independent variable; *ε_i_* represents residuals; $\left(u_{i},v_{i}\right)$ is the spatial coordinate of the *i*-th sample point; and $a_{k}(u_{i},v_{i})$ is the value of the continuous function at the point *i*. The continuous function $a(u_{i},v_{i})$ is defined as,

(5) $$a(u_{i},v_{i})={\left(X^{T}W(u_{i},v_{i})X\right)}^{-1}X^{T}W(u_{i},v_{i})y$$

where $W(u_{i},v_{i})$ is the distance weight matrix, which is a diagonal matrix with diagonal elements $\left(W_{i1},W_{i2},\ldots ,W_{in}\right)$. The non-diagonal elements are 0, *n* is the sample size; and $W_{ij}$ is the impact of point *j* to point *i* and is always defined as $\exp(-d_{ij}^{2}/h^{2})$, where d*_ij_* is the distance between point *i* and point *j* and *h* is a custom bandwidth. The R^2^ value, R_c_^2^, Akaike information criterion (AIC) values were computed for each model to assess the goodness-of-fit.

## 3. Results and Analysis

### 3.1. Descriptive Analysis

**Figure 4:**
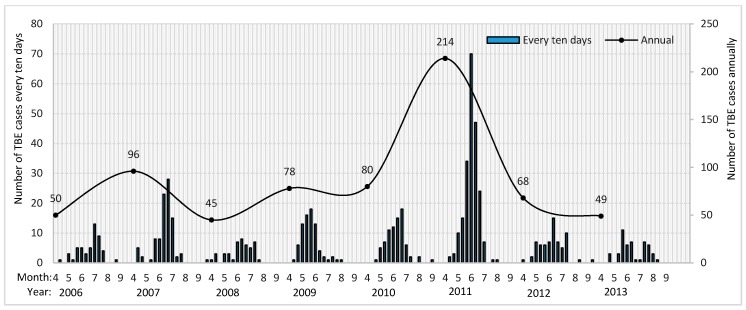
Annual and every-ten-days number of tick-borne encephalitis (TBE) cases from 2006 to 2013.

A total of 675 TBE cases were reported in 13 counties during 2006–2013 in Hulunbuir city. The annual and every-ten-days variations of the TBE cases are shown in Figure 4. The reported TBE cases were below 100 during most years. However, the case number increased to 214 in 2011. The annual TBE cases were reported only from April to September, which indicates that this type of disease is characterized by a high number of incidences in spring and summer. To be more specific, the number of TBE cases increased rapidly from late April to early May. The reported case number reached its peak value in the beginning of June. The case number decreased after that. In mid-July, the case number was ≤10. The number of TBE cases decreased after August and was small.

During these 8 years, there were 490 male TBE cases and 185 female TBE cases. The ratio of male to female cases is ~2.6 during the eight years. The statistical data show that the number of male cases is much higher than that of female cases. Taken the aging group and the occupation into consideration, there were 176 cases aging from 30 to 39, accounting for 26.1% of total number, and 251 cases aging from 40 to 49, accounting for 37.2% of total number. The statistical professional data showed that, among the 481 cases which have registered their occupation, there were 208 workers, accounting for the highest proportion. The statistical results indicated that people aging from 30 to 50 who work more often in forest, such as lumbermen, are infected more easily with TBE. 

### 3.2. Spatial Autocorrelation

The local spatial autocorrelation results were shown in Table 1. A significant positive spatial autocorrelation of the TBE case distribution was observed during the study period. The global Moran’s *I* values range from 0.079 to 0.144 and are much greater than the expected value (−0.008). Except for 2012, the *p*-value is greater than 0.1. The *p*-value of 2006 and the average of the eight years is smaller than 0.01. In most years, the spatial distribution of TBE cases is thus very likely spatially aggregated.

**Figure 5:**
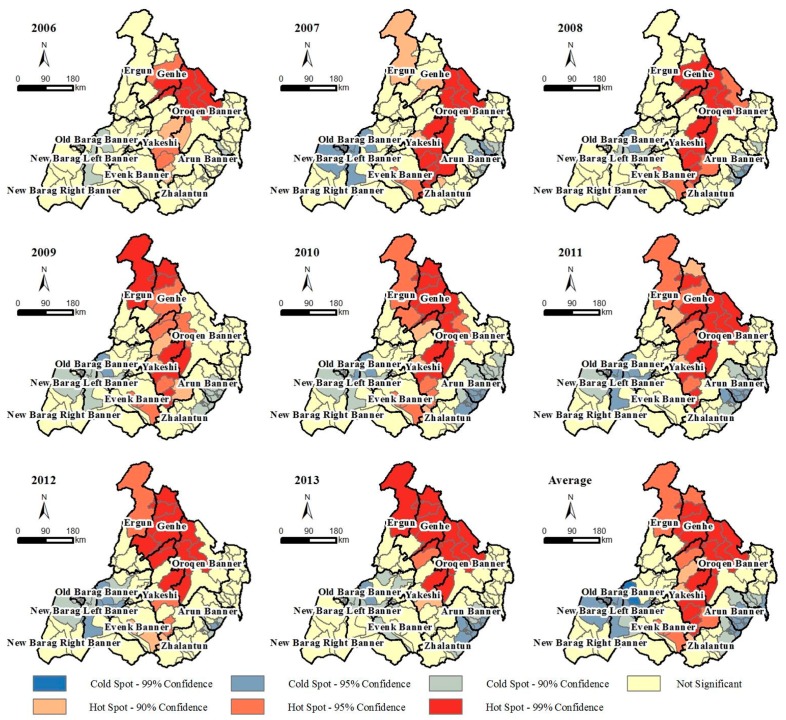
Annual and average local indicators of the spatial association cluster maps for the tick-borne encephalitis (TBE) cases in Hulunbuir from 2006 to 2013.

The annual and average spatial local autocorrelations of the TBE case number in Hulunbuir from 2006 to 2013 are shown in Figure 5. The TBE high-risk accumulation area is mainly concentrated in central Hulunbuir and the low-risk areas are mainly distributed in the most eastern and western parts of Hulunbuir. The high-risk aggregations change almost every year. In 2006, the TBE transmission hotspots were mainly concentrated in the northern Greater Khingan Range including several towns of the Genhe and Oroqen counties. From 2007 to 2009, the hotspot region expanded along the mountain range to the northern and southern areas. The hotspot area extended from the most northern Mordaga County to the most southern Yimin Sumu until 2010. In 2011 and 2012, the hotspot area spread to the east and west. In 2013, the hotspots mainly concentrated in the center and northern Hulunbuir. The local spatial autocorrelation distribution of the average case number during eight years indicates that the central Greater Khingan Range was the TBE transmission hotspot. 

### 3.3. Global Regression Analysis

The correlation coefficients between the geographic factors and TBE incidences are shown in Table 2. All *p*-values are below 0.01, which indicates that all factors significantly correlate with the TBE incidence number and are included in the regression operations.

However, the data ranges of RH (57.73, 67.06) and Prep (18.57, 45.02) were too small, which introduced strong spatial clusters in the regression process. When performing the GWR method by using RH and Prep in ArcGIS, there always be error warnings. So in this paper, the transformed forms of these two variables were used in the regression models to increase the value variations and ensure the normal operation of GWR in ArcGIS. The transformed forms of RH and Prep were (RH − 57) × 10,000 and (Prep − 18) × 10,000, respectively.

The stepwise MLR results are shown in Table 3. The regression yielded 12 equations. The last equation was verified to be the optimal, with a relatively low F-value, the biggest R^2^ value, and a relatively low correlation between the factors. 

Table 4 shows the standard coefficients of the optimal MLR model. The standardized optimal MLR model is as follows:

(5) $$\mathrm{lg}(R_{TBE})=0.133Z_{Slope}-0.064Z_{DEM}+0.897Z_{NDVI}-0.379Z_{EVI}-1.376Z_{Temp}-1.041Z_{RH}+0.236Z_{Prep}-0.145Z_{PF}$$

where $\mathrm{lg}(R_{TBE})$ is the logarithmic EBS smoothed TBE incidence. All *Z* variables are the standardized independent variables. The standardized coefficients reflect the impact of the variables on the TBE risks. The standardized coefficients can be arranged in the following descending order: temperature (|*Z*| = 1.376), RH (|*Z*| = 1.041), NDVI (|*Z*| = 0.897), EVI (|*Z*| = 0.379), precipitation (|*Z*| = 0.236), precipitation frequency (|*Z*| = 0.145), slope (|*Z*| = 0.133), and DEM (|*Z*| = 0.064). The impact degree of these factors has the following descending order: temperature, RH, vegetation coverage, precipitation, and topography.

### 3.4. Local Regression Analysis

The correlation statistics between the factors mentioned in Section 3.3 show that the RH and temperature are negatively correlated; the correlation coefficient is −0.89. The precipitation and precipitation frequency are positively correlated; the correlation coefficient is 0.60. The NDVI and EVI are positively correlated; the correlation coefficient is 0.95. Not only are the values of these three pairs related but also the geographic characteristics. To separate the strong multicollinearity between these factors, the factors were combined individually to model the GWR. The precipitation frequency is significantly auto correlated; hence, it was not included in the model. The combination of factors with the best fitting effect was selected as the optimal GWR model. The GWR fitting effects of different combinations of the geographic and environmental factors are shown in Table 5. Model 1 comprises the DEM, slope, aspect, precipitation, NDVI, and RH and has the highest R^2^ and R_c_^2^ value, and lowest AIC value.

**Figure 6:**
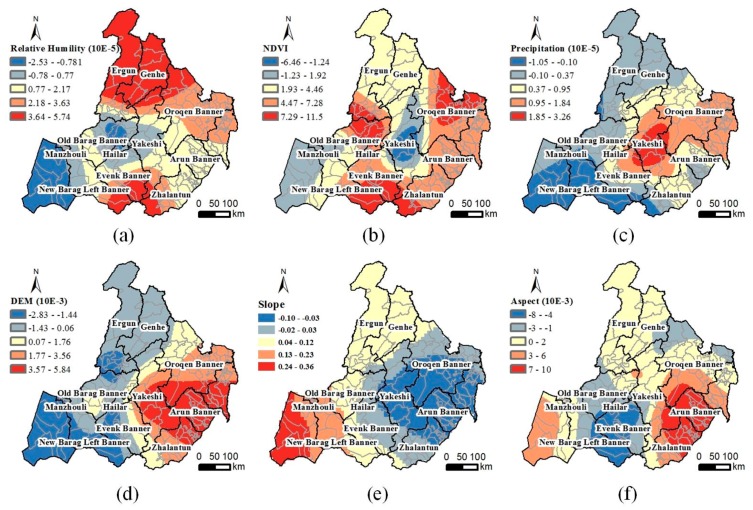
Spatial distribution of the GWR model coefficients: (**a**) relative humidity, (**b**) NDVI, (**c**) precipitation, (**d**) DEM, (**e**) slope, and (**f**) aspect.

The spatial distribution of the different coefficients of the variables in the calculated GWR model is shown in Figure 6. The spatial distribution of the correlation between the TBE risks and different geographic and environmental variables is quite different:

(1) Relative humidity: The RH in the three northern counties and several southern counties and the TBE risk are positively correlated (Figure 6a). The TBE risk in these areas increases with the RH. The TBE risk in the New Barag Right Banner and the RH are negatively related. Regions with relatively high RH values always have a lower risk.
(2) Vegetation index: Based on the spatial distribution of the NDVI coefficient in Figure 6b, the TBE risk is positively correlated with the NDVI in the Old Barag Banner, Oroqen Banner, and several southernmost towns. The TBE risk in these areas increases with increasing vegetation cover. In the middle Yakeshi County, especially in Wunu’er and Miandu, the TBE risk reaches the highest value while it is negatively correlated with the NDVI. No direct relationship between the increasing vegetation coverage and TBE risk was observed in these areas.
(3) Precipitation: The TBE risks in southwestern Hulunbuir are negatively correlated with the precipitation (Figure 6c). The TBE risks in this area decrease with increasing precipitation. The TBE risks and precipitation in the center of the Yakeshi County are positively correlated. The risks increase with increasing precipitation.
(4) DEM: The correlation between the TBE risks and DEM changes from negative to positive from west to east (Figure 6d). Areas with high elevation in western Hulunbuir exhibit less risk than the low regions. The effect of the DEM is the opposite in eastern Hulunbuir.
(5) Slope: The correlation between the slope and TBE risk is negative in the east, while it is highly positive in the west. In western Hulunbuir, where most of the land cover is grassland, the TBE risk is a bit higher at a steep slope than in gentle areas. In contrast, the TBE risk in the broad farmland of eastern Hulunbuir is lower at a steep slope than in the gentle areas (Figure 6e).
(6) Aspect: The TBE risk in northern Hulunbuir is slightly impacted by the change of aspect. In contrast, there are two different situations in the southern area. The TBE risk is negatively correlated with the aspect in the Evenk and New Barag Right banners, while they are positively correlated in the southeastern Arun Banner and Zhalantun County (Figure 6f).

## 4. Discussion

### 4.1. Endemic Seasonal Features of TBE

The descriptive analysis resulted in the seasonal high incidences of TBE. This feature was highly consistent with previous studies. The seasonal feature of people infected by TBE virus is likely to be highly correlated with the epidemiological process [29]. The virus carried by the adult female ticks are more likely to spread to humans from spring to fall when the nymphs develop into adults to search for large mammals as the dead end hosts e.g., humans and roe deer [17]. In the northeastern part of China, *Ixodes persulcatus* began to be active at early April, when the daily average temperature reached 0 °C. The tick density reached a peak at May. The number of active ticks decreased during June and July, when daily average temperature rise to 20 °C. Until mid and end of September, the daily average temperature dropped below 10 °C, and ticks were being not active [6]. Adding the delayed incubation period of human beings, usually 7 to 14 days, the reported annual prevalence time of TBE, mentioned in Section 3.1 is quite similar to the active cycle of local ticks.

### 4.2. Predicted Risk Distribution

**Figure 7:**
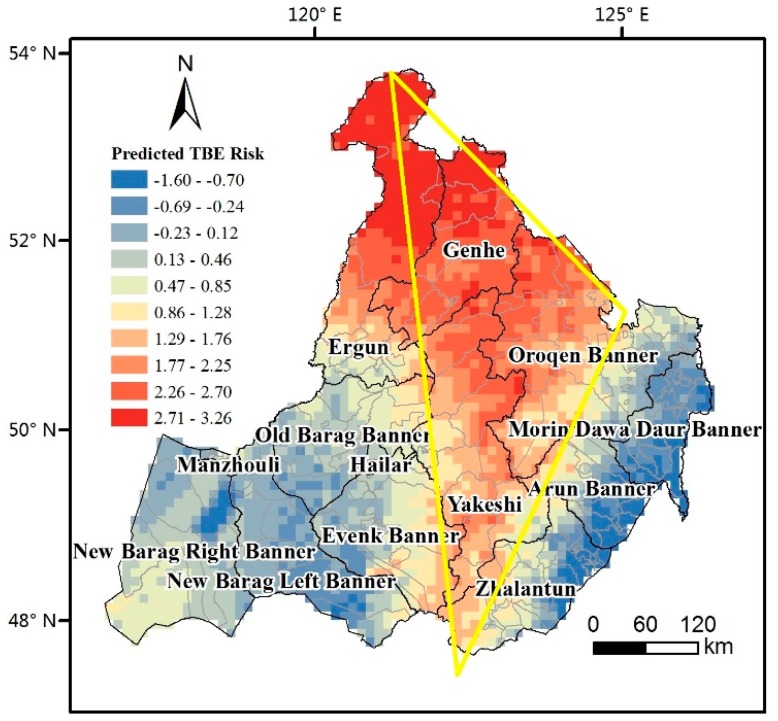
Spatial distribution of the predicted tick-borne encephalitis (TBE) risk. The background color is the predicted result of the Geographic Weighted Regression model based on the TBE risks.

Figure 7 shows the predicted result of TBE risks based on the first model in Table 5. The distribution of the TBE risks is notably diverse in Hulunbuir. Central Hulunbuir is characterized by a high-risk triangle zone. The north angle of this triangle is located in the virgin forest of the Great Khingan Mountains. The triangle expands along the border of China and Russia to the northwestern Oroqen Banner, which is the eastern angle of the high-risk triangle. The southern angle reaches the most southern point of Zhalantun County. The specific characteristics of the TBE risk are as follows:

#### 4.2.1. Central High-Risk Triangle Zone

From north to south, the high-risk triangle covers Genhe, Oroqen, and Yakeshi. The high-risk zone is bounded by the boundary line of the eastern side of the Khingan Range and the Northeast Plain in the east; the western side is bounded by the Hulunbuir Prarie. The southernmost point is located in the deciduous coniferous forest of Taerqi, Yakeshi.

The combination with the spatial distribution of the different coefficients of the variables of the local regression model results shows that the correlations between the TBE risks and geographic and environmental factors change depending on the spatial location. The risk of most of the triangle area increases with the increase of the RH, especially at the northern and southernmost points; the correlation between the TBE risks and RH is the highest. The risk increases by 3.64 to 5.74 for each 0.1-increase of the RH, which is consistent with previous studies [30,31,32]. However, this correlation becomes smaller from the apex angles to the middle part in central Yakeshi and even transforms into a negative correlation. With respect to NDVI, the correlation in most parts of the triangle area appears to be positive. For example, the TBE risk in northern Ergun and parts of the Genhe Counties increases by 0.19 to 0.45 for each 0.1-increase of the calculated NDVI. The risk in most locations of this triangle is likely positively correlated with the precipitation. When the precipitation in central Yakeshi increases by 1 mm, the TBE risk also increases by 1.85 to 3.26. With respect to topography, the TBE risk in most of the triangle region is positively correlated with the aspect. The correlation with the DEM and slope seems to be quite different in the northern and southern areas. The TBE risk is negatively correlated with DEM in the northern triangle area; however, it is positively correlated with the slope. When the elevation increases by 1000 m, the risk accordingly decreases by 1.77 to 5.84. When the slope increases by 10°, the risk also increases by 0.3 to 1. When the elevation in the central and southern triangle area increases by 1000 m, the TBE risk increases by 1.77 to 5.84. When the slope increases by 10°, the TBE risk decreases by 0.5 to 1.4.

#### 4.2.2. Western Low-Risk Belt

Western Hulunbuir features a low TBE risk, which is mainly distributed at the western side of the Great Khingan Range, the broad prairie. Although the TBE risk of the western belt is lower than that of the center triangle area, the risk differs depending on the geographic distribution. The TBE risk of the eastern strip near the Great Khingan Mountains and most of the western New Barag Right Banner is slightly higher than that of the central belt. 

The TBE risk in the New Barag Right Banner is negatively correlated with the RH. Once the RH increases to 0.1, the TBE risk decreases by 0.78 to 2.53. In contrast, the TBE risk of the eastern strip, next to the central high-risk triangle area, is positively correlated with the RH; a increment of 0.1 of the RH leads to a TBE risk increase of 3.64–5.74. With respect to the vegetation factor, the TBE risk in most parts of the western belt is positively correlated with NDVI, except in the New Barag Right Banner. The TBE risk of the southwestern New Barag Right and New Barag Left banners decreases by 0.78–2.53 with a precipitation increase increment of 1 mm. The correlation between the TBE risk and precipitation in parts of the northeastern Evenk Banner is quite similar to that in Yakeshi. The topography of most parts is negatively correlated with the DEM. At 1000-m elevation increments, the TBE risk decreases by 2.83 at most. The TBE risk of most of the western low-risk belt is positively correlated with the slope; at 1°-increments, the TBE risk increases by 0.03–0.28.

#### 4.2.3. Eastern Low-Risk Belt

The TBE risk is relatively low in eastern Hulunbuir. The TBE risk decreases from the eastern side of the Great Khingan Range to the eastern border of Hulunbuir. It seems that the farther the location is away from the mountains in this belt, the lower is the TBE risk.

Most of the geographic and environmental factors in the eastern low-risk belt, such as the RH, NDVI, precipitation, DEM, and slope, are positively correlated with the TBE risk. In detail, the RH, precipitation, and NDVI show stronger correlations with the TBE risk in the southern and northernmost areas than in the central belt. In contrast, the DEM and slope exhibit weaker correlations with the TBE risk in the southern and northernmost areas than in the central belt.

The results of the global regression analysis show that the temperature and relative humility more likely reflect the distribution of the TBE risk. These results are consistent with previous studies, which reported the strong influence of temperature and moisture on the distribution of ticks and tick-borne diseases. However, the GWR results add the spatial variety to the relationship. In particular, the impact of the relative humility in the most northern and southern areas is the greatest. The influence of DEM is not always positive. The higher the area in northern Hulunbuir is, the lower is the risk. 

### 4.3. Regression Models

Regression models were used to simulate the spatial risks of some certain diseases, especially natural focus diseases, based on the quantitative relations between factors. According to whether or not the spatial effect was considered, the regression models can be divided into classic statistical regression models and spatial regression models. The classic regression models include ordinary least square (OLS), generalized linear model (GLM) and generalized linear model (GLM), time series Poisson regression model, and Logistic regression model, etc. [33]. Time series Poisson regression was performed taking into account seasonality, lag effects and long-term trends [34]. It was often used in the detection of the relationship between the natural focus disease and climate factors [34,35]. Logistic regression was frequently used in the study of diseases with little number of cases [36,37,38]. Using stratified variables to replace numeric variable is more easily to reveal the relationship between predictive factors and involved factors in these kind of disease. The most widely used spatial regression model is GWR, which explicitly account for spatial heterogeneity. The strength of GWR is that it allows for the creation of a map showing the continuous surface of parameter values and an examination the spatial heterogeneity of these parameters [28]. Combined with spatial correlations and spatial heterogeneity, GWR is better to reflect more accurate spatial changes in the spatial distribution of disease risk [39]. Previous studies have used this method in the study of tick-borne diseases [30,40].

However, the results of GWR comes along with some problems. For one thing, there would be a deviation between the predicted risks and observed values in partial area. For example, in Kerlun town, southwestern Hulunbuir, the predicted risk ranged from 0.37 to 1.03, which was relatively lower than the transformed TBE incidence value (1.92) compared with surrounding towns. Based on the geographic weights calculation, the regression process of one unit was associated with its adjacent units. The similar values of selected factors in southwestern part of Hulunbuir induced the deviant predictions. For another, the spatial distribution of GWR coefficients, which shows the spatial variability of parameter estimates, would be biased to show the correlation between the predicted risk and a specific single factor. Because the distribution of predicted TBE risks was formed by multi-factors and their interactions. Even though, combined with the impacted degree of some main factors on TBE risks referring to the results of global regression calculation, the spatial distribution of the coefficients of relative humidity, vegetation index and precipitation in GWR still brought out the spatial variation of these main factors to TBE, and disclosed the impact of these geographical factors to TBE risks in different area.

## 5. Conclusions

We propose an effective method to assess the spatiotemporal distribution of the TBE risks and related geographic and environmental factors. Based on Hulunbuir, the Chinese area most seriously affected by natural TBE, the spatial characteristics of the TBE epidemiology were analyzed using spatial and temporal analysis. We obtained preliminary and global results about the impact of the referenced factors on the TBE risk using MLR and assessed the spatial distribution of the TBE risk by analyzing the GWR results. The conclusions are as follows:

(1) The spatial autocorrelation results show that the distribution of the TBE risk in Hulunbuir was significantly autocorrelated from 2006 to 2013. The high-risk aggregation area gradually changes during the study period. The high-risk TBE aggregation area first extends from the northern part of the Great Khingan Range southward. The aggregation foci return back to the origin in 2011, and the high-risk aggregations continue to expand northward up to Moerdaoga, Ergun County. The statistical data show that the people in Hulunbuir more easily get infected with TBE in spring and summer. The prevalence of the patients has notable occupational and gender characteristics. Male workers inhabiting or working in forests more easily get infected.
(2) The impact degree of the geographic and environmental factors on the TBE risk has the following descending order: temperature, RH, vegetation coverage, precipitation, and topographic information. The temperature and RH in Hulunbuir are strongly negatively correlated. In addition, the spatial distribution of the different coefficients of the variables in the local regression model show that the correlations between the TBE risk and geographic and environmental factors change depending on the spatial location.
(3) The distribution of TBE risk in Hulunbuir was quite particular. Central high-risk region seemed to be a triangle area. The eastern and western belts are at low TBE risk. The high-risk triangle includes Ergun, Genhe, Oroqen Banner, and Yakeshi County. The TBE risk inside the triangle region increases from south to north. The most relevant factor in this triangle is the RH. The TBE risk in most parts of this triangle is positively correlated with NDVI, precipitation, and aspect and negatively correlated with slope; they are negatively correlated in the northern triangle. The local regression results provide a risk evaluation model and data support for the TBE prediction and control.

Still, there are some research limitations in this study. For instance, only average values of TBE incidence and related factor were used to do the temporal analyses in this paper. In fact, the geographical and environmental factors are correlated with TBE risks in a more accurate time scale. Changes in the geographical and environmental factors influence the risk of TBE through impact on the life cycle dynamics of the tick, the tick habitat, and the host animals, as well as through changes in human behavior and human-vector-host animal interactions [17,41,42]. As a reference, Wang et al. made detailed contrast experiment with a set of fifty replicate, stochastic 208-week (4-year) simulations to detect the simulated effects of changes in host density on spatial-temporal prevalence of infected ticks [43]. To further explore the correlation of TBE disease case occurrence with the environmental factors and find real reasons for the changes of TBE hot spots, data of vectors and vertebrate hosts will be added into the study with more accurate time scale. 

The human activities factor is also a quite noticeable factor to the assessment of TBE risks. This paper mainly focused on the impact of physical geographical and environmental factors on TBE risks. Limited by data sources, the study of human population was not taken too much into this paper. Human factors involves the vaccination and immunity conditions, emigration situation, and land cover, etc. By continuous cooperation with epidemic departments and other related institutes, in future study, human factors will be included for further detection of TBE risks.

## Figures and Tables

**Table 1:** Spatial autocorrelation analysis of the annual tick-borne encephalitis (TBE) cases in Hulunbuir from 2006 to 2013.

Indices	2006	2007	2008	2009	2010	2011	2012	2013	2006–2013 Average
Moran’s *I*	0.156	0.113	0.092	0.089	0.110	0.106	0.079	0.115	0.144
*E(I)*	−0.008	−0.008	−0.008	−0.008	−0.008	−0.008	−0.008	−0.008	−0.008
*Z*-score	3.205	2.085	1.791	1.707	2.169	2.003	1.485	2.081	2.655
*p*-value	0.001	0.037	0.073	0.088	0.030	0.045	0.137	0.037	0.008

**Table 2:** Correlation coefficients between the tick-borne encephalitis (TBE) incidence number and geographic factors.

Elements	Aspect	Slope	DEM	EVI	NDVI	Prep	PF	SH	RH	Temp
Coefficients	0.15	0.56	0.46	0.46	0.63	0.28	0.68	−0.40	0.60	−0.60
*p*-value	<0.01	<0.01	<0.01	<0.01	<0.01	<0.01	<0.01	<0.01	<0.01	<0.01

DEM, digital elevation model; EVI, Enhanced Vegetation Index; NDVI, Normalized Difference Vegetation; Prep, precipitation; PF, precipitation frequency; SH, sun hours; RH, relative humidity; Temp, temperature.

**Table 3:** Multiple linear regression models and test indicators.

Model Code	Factors	*F*-Value	*p*-Value	R²	R_c_²
1	PF	1.457E3	0.000	0.384	0.384
2	PF, Slope	916.339	0.000	0.440	0.439
3	PF, Slope, Temp	831.242	0.000	0.516	0.516
4	PF, Slope, Temp, NDVI	670.466	0.000	0.535	0.534
5	PF, Slope, Temp, NDVI, RH	609.865	0.000	0.566	0.566
6	PF, Slope, Temp, NDVI, RH, EVI	527.187	0.000	0.576	0.574
7	Slope, Temp, NDVI, RH, EVI	632.140	0.000	0.575	0.574
8	Slope, Temp, NDVI, RH, EVI, Aspect	529.974	0.000	0.577	0.576
9	Slope, Temp, NDVI, RH, EVI, Aspect, DEM	455.911	0.000	0.578	0.577
10	Slope, Temp, NDVI, RH, EVI, Aspect, DEM, Prep	401.706	0.000	0.580	0.578
11	Slope, Temp, NDVI, RH, EVI, Aspect, DEM, Prep, PF	358.195	0.000	0.580	0.579
12	Slope, Temp, NDVI, RH, EVI, DEM, Prep, PF	402.405	0.000	0.580	0.579

RH, relative humidity; Temp, temperature; Prep, precipitation; PF, precipitation frequency; and SH, sun hours.

**Table 4:** Coefficients of the optimal model.

Independent Variables	Coefficient	Standard Coefficient	*t*-Value	*p*-Value
Constant	15.336		11.040	0.000
Slope (°)	0.026	0.133	4.548	0.000
DEM (km)	−0.207	−0.064	−2.337	0.020
EVI	−3.978	−0.379	−7.723	0.000
NDVI	5.148	0.897	14.969	0.000
PF (days)	−0.006	−0.145	−2.546	0.011
Prep (mm)	0.022	0.236	4.475	0.000
Temp (°C)	−0.449	−1.376	−14.604	0.000
RH (%)	−0.263	−1.041	−11.111	0.000

RH, relative humidity; Temp, temperature; Prep, precipitation; PF, precipitation frequency; and SH, sun hours.

**Table 5:** Geographic Weighted Regression fitting effects of different combinations of geographic and environmental factors.

Model ID	Involved Factors	R^2^	R_c_^2^	AIC
1	DEM, Slope, Aspect, Prep, NDVI, RH	0.98	0.99	7.55
2	DEM, Slope, Aspect, Prep, EVI, RH	-	-	-
3	DEM, Slope, Aspect, Prep, NDVI, Temp	0.87	0.88	56.85
4	DEM, Slope, Aspect, Prep, EVI, Temp	0.96	0.96	24.46

RH, relative humidity; Temp, temperature; Prep, precipitation; PF, precipitation frequency; and SH, sun hours.
